# Immune Check Point Inhibitors and Immune-Related Adverse Events in Small Cell Lung Cancer

**DOI:** 10.3389/fonc.2021.604227

**Published:** 2021-03-18

**Authors:** Wanting Hou, Xiaohan Zhou, Cheng Yi, Hong Zhu

**Affiliations:** Department of Medical Oncology, Cancer Center, West China Hospital, Sichuan University, Chengdu, China

**Keywords:** small cell lung cancer, immune check point inhibitors (ICIs), immune-related adverse events (irAEs), programmed cell death protein 1 (PD-1), programmed cell death ligand protein 1 (PD-L1), cytotoxic T lymphocyte-associated antigen 4 (CTLA-4)

## Abstract

Small cell lung cancer (SCLC) is a malignant solid tumor. In recent years, although immune check point inhibitors (ICIs) have achieved important advances in the treatment of SCLC, immune-related adverse events (irAEs) have occurred at the same time during the therapeutic period. Some irAEs lead to dose reduction or treatment rejection. The immune microenvironment of SCLC is complicated, therefore, understanding irAEs associated with ICIs is of great importance and necessity for the clinical management of SCLC. However, the lack of comprehensive understanding of irAEs in patients with SCLC remains remarkable. This review aims to provide an up-to-date overview of ICIs and their associated irAEs in patients with SCLC based on present clinical data.

## Introduction

Lung cancer has jeopardized the health of millions of people worldwide (1). Histologically, lung cancer is usually classified into non-small cell lung cancer (NSCLC) and small cell lung cancer (SCLC). Although NSCLC is diagnosed more often, SCLC is much more likely to be associated with a worse prognosis. SCLC is characterized by rapid growth, early metastasis, and frequent relapse (2) and its diagnoses are further sub-divided into limited-stage SCLC (LS-SCLC) or extensive-stage SCLC (ES-SCLC). LS-SCLC is diagnosed when the diseased region is confined to one hemithorax within a tolerable radiation field, while ES-SCLC is diagnosed when the disease has spread beyond one hemithorax. The prognosis of patients with LS-SCLC is better than those with ES-SCLC, the survival time of patients with LS-SCLC ranges from 15 to 20 months and their 5-year survival rate is ~20–25%. In comparison, the survival time of patients with ES-SCLC ranges from 8 to 13 months, with a 5-year survival rate of only about 2% (3, 4). Unfortunately, ~70% of SCLC patients are diagnosed with ES-SCLC (5).

Compared with NSCLC, SCLC's effective therapy regimens are limited. In the past, platinum-based chemotherapy has been the cornerstone of the SCLC therapeutic landscape. The overall response rate (ORR) of ES-SCLC patients receiving first-line chemotherapy is ~67%, with most patients showing resistance to chemotherapy within a short time and overall survival (OS) of <1 year. Less than 30% of patients are diagnosed with LS-SCLC and they respond well to chemotherapy (ORR: 82–87%). However, preventing relapse and progression is still challenging (6). At present, second-line treatment options for patients with relapsed SCLC are limited. In 2007, the FDA approved topotecan as a second-line treatment option for SCLC. The response rate to topotecan of patients who have relapsed is 20–25%, with a 1-year survival rate of 10–30% (7, 8). Over the years, there are a few new explorations in the therapeutic landscape of SCLC. The success of immunotherapy for NSCLC is great inspiration for SCLC therapy. Several clinical trials on monoclonal antibodies targeting programmed cell death ligand protein 1 (PD-L1), programmed cell death protein 1 (PD-1), and cytotoxic T-lymphocyte-associated antigen 4 (CTLA-4) have been or are currently being conducted for SCLC. The response rate to immunotherapy is lower for SCLC than for other tumors such as NSCLC and melanoma, as a possible result of the lack of biomarkers for choosing beneficial populations. Nevertheless, immunotherapy still brings breakthroughs for SCLC therapy. Based on the encouraging results of CheckMate032, KEYNOTE028, and KEYNOTE158, FDA approved nivolumab and pembrolizumab monotherapy as third or later line for the treatment of patients with relapsed SCLC. Compared with chemotherapy alone, the PD-L1 inhibitors atezolizumab and durvalumab plus the chemotherapy agent's platinum and etoposide have demonstrated prolonged OS among ES-SCLC patients. In addition, these two PD-L1 inhibitors plus chemotherapy have been approved by FDA as first-line therapy for ES-SCLC (Table 1). Recently, several meta-analyses analyzed different first-line treatments for ES-SCLC patients and demonstrated that PD-L1 inhibitors durvalumab and atezolizumab plus etoposide-based chemotherapy may be the best choice as first-line therapy for ES-SCLC patients (9–11).

**Table 1:** FDA approved ICIs in SCLC.

**Agent**	**Target**	**Therapy line**	**Patients**	**Approve time**	**Based clinical trail**
Nivolumab	PD-1	Third line or later line	Relapsed-SCLC	2018.08	CheckMate032
Atezolizumab+ platinum-etoposide	PD-L1	First line	ES-SCLC	2019.03	IMpower133
Pembrolizumab	PD-1	Third line or later line	Relapsed-SCLC	2019.06	KEYNOTE028 and KEYNOTE158
Durvalumab+ platinum-etoposide	PD-L1	First line	ES-SCLC	2020.03	CASPIAN

Meanwhile, there is increasing concern regarding immune-related adverse events (irAEs) of ICIs (12–14). The appearance of some irAEs have beenshown to be related to the efficacy of ICI agents in patients with NSCLC and melanoma (15, 16), but this relationship has not been established in patients with SCLC. SCLC is characterized by complex immunophenotypes, and autoimmune-related paraneoplastic syndromes are commonly reported among SCLC patients (17). Understanding irAEs of ICIs is crucial for the clinical management of SCLC and for further improvement of the immunotherapeutic approach to SCLC, but few studies have focused on irAEs in SCLC recently. Therefore, in this article, we present an up-to-date review of ICIs and irAEs in SCLC based on data from present clinical trials.

### Mechanism of ICIs and irAEs

**Figure 1:**
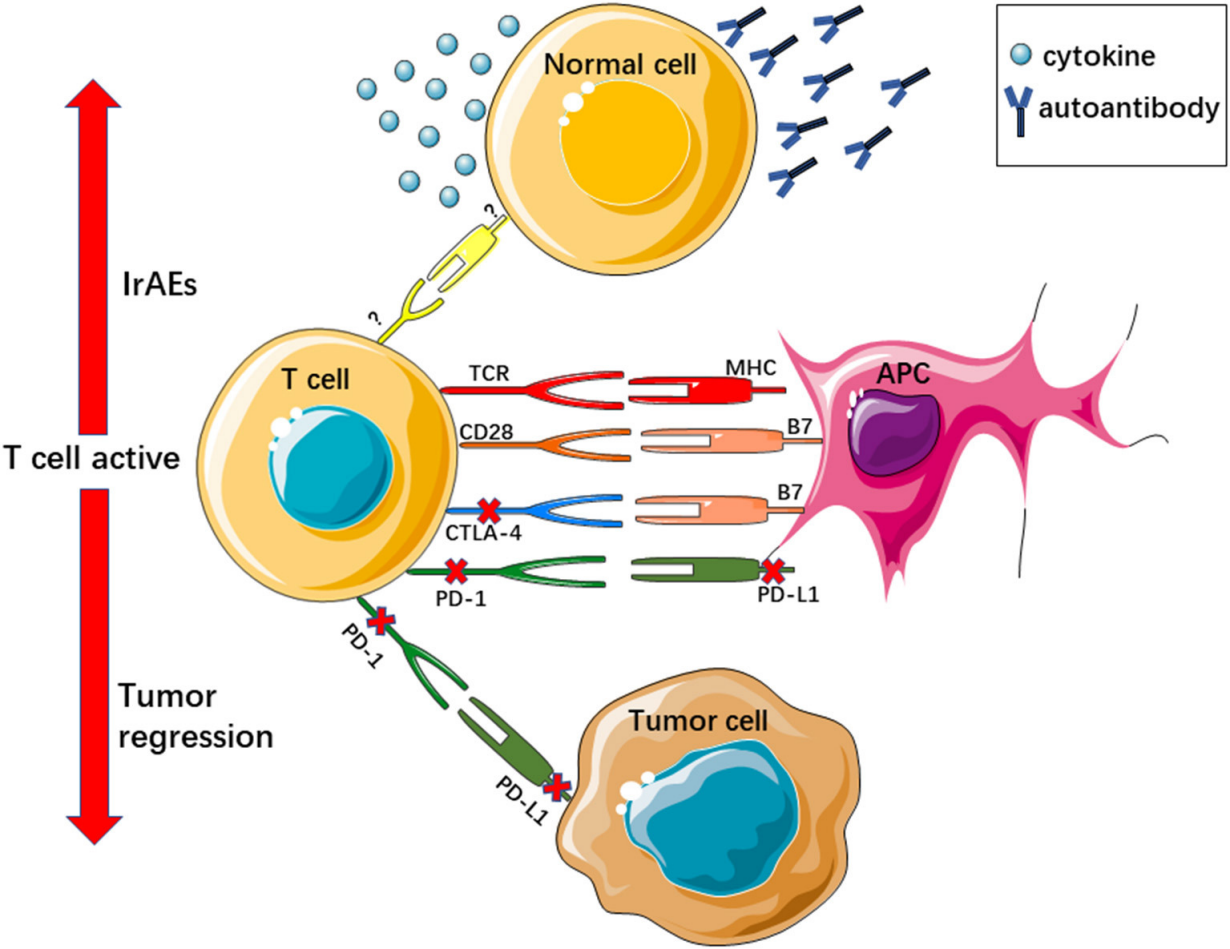
ICIs reactive T cell active regress tumor growth and evoke irAEs. TCR, T cell receptor; MHC, major histocompatibility complex.

Under normal conditions, immune inhibitor molecules such as CTLA-4 and PD-1/PD-L1 function as negative regulators and maintain the balance of the immune system. CTLA-4 and CD28 are commonly expressed on the surface of T cells, where they compete with the same binding sites as CD80/CD86 on the surface of antigen-presenting cells (APCs). CD28 combines with CD80/CD86 to provide an activation signal for T cells. The combination of CTLA-4 and CD80/CD86 impedes T-cell activation and downregulates T-cell responses (18, 19). Unlike CTLA-4, PD-1 is expressed on the surface T cells or other immune cells, and its ligand, PD-L1, is expressed on the surface of APCs and other immune cells. Tumor cells can also express PD-L1, and PD-1 and PD-L1 combine on the surface of tumor cells, leading to downregulation of the T-cell response, and helping tumor cells to escape from the host immune response (20, 21). Tumor cells upregulate these immune inhibitor molecules to evade the immune system, resulting in tumor initiation, progression, and metastasis. The ICI blockade of PD1/PD-L1 or CTLA-4 activates the body's antitumor immunity (Figure 1).

IrAEs also occur in this context. IrAEs are generally considered to be related to the damage of normal tissues, which results from immunotherapy, and immune tolerance is affected by ICIs, resulting in activated T cells targeting non-tumor antigens or self-peptides. IrAEs affect almost all organs. The most common irAEs include rash, pruritus, colitis, hypothyroidism, hyperthyroidism, and pneumonitis. The precise pathophysiology of irAEs is still undefined. Existing studies suggest that autoantibodies play an important role in irAEs. Some autoantibodies may have cross-reactivity with antibodies during immunotherapy (22). One example is vitiligo, which is caused by an autoantibody attack on melanocytes, and it is also frequently observed among melanoma patients who have received ICI therapy (23). In addition, some cytokines may be involved in irAEs (22). For instance, the levels of the interleukin-17 were obviously higher in patients with ipilimumab-related colitis (24). Host factors, intestinal microbiota, genetic risk factors, and specific antigen exposures may all be involved in irAEs (25). Viruses or co-administered drugs can also provoke irAEs (26). CTLA-4 and PD1/PD-L1 inhibitors usually display different irAEs. Reportedly, the occurrence rate of diarrhea and colitis in patients administered CTLA-4 is higher than that in those administered PD-1/PD-L1 antibodies. Thyroiditis and pneumonitis are more commonly observed in patients who received PD-1/PD-L1 inhibitors. Compared with ICI monotherapy, combinations of ICIs increase the risk of irAEs. Tumors also influence irAEs, with colitis and skin irAEs being more common in patients treated with ICIs for melanoma and pneumonitis occurring more frequently in lung cancer patients treated with ICIs (25). Accurately recognizing irAEs and closely following up patients who have used ICIs are essential parts of immunotherapy.

### The Immune Characteristics and Response to ICIs of SCLC

The efficacy of immunotherapy is largely determined by the internal immune microenvironment of the tumor (27). However, it is widely regarded that SCLC has a unique and complex immune microenvironment.

Consistently, previous studies regard SCLC as a kind of immunogenic cancer) One of the hallmarks of SCLC is high tumor mutational burdens (TMBs) (28, 29), which are usually used as a predictor of ICI efficacy for many cancer types, including NSCLC (30) and melanoma (31). Reportedly, High TMBs could influence further neo-antigens to activate the immune system (32). Moreover, paraneoplastic syndromes are commonly observed in SCLC patients and are mediated by autoantibodies. Evidence shows that SCLC patients with neurologic paraneoplastic syndromes present with better prognosis (33, 34). While immunosuppression does exist in SCLC, lower expression of class I major histocompatibility antigens, tumor-infiltrating lymphocytes, and PD-L1 have also been reported in some SCLC patients (3).

The response rate to ICIs, particularly ICI monotherapy, was lower in most patients with SCLC than in those with other tumors. PD-1 antibody nivolumab monotherapy did not present any advantage in the improvement of OS or progression-free survival (PFS) as second-line treatment setting for relapsed SCLC according to the Checkmate 331 study (35). Moreover, the PD-L1 inhibitor durvalumab monotherapy for relapsed SCLC patients presented a confirmed ORR of only 9.5% (36).

Furthermore, the overall low response to ICIs of SCLC patients may be due to the lack of biomarkers. In the past years, relatively few tumor specimens for SCLC have been available. In addition, immune heterogeneities have been found among SCLC patients; in particular, tumor cell PD-L1 expression was different among various studies (37). No reliable biomarkers have yet been confirmed in terms of the population of SCLC patients who will benefit from ICI immunotherapy, which could influence the ORR. Recently, comprehensive research related to SCLC's biomarkers in the tumor stromal cell or in the blood are largely being conducted (38, 39). However, the findings still need to be assessed in large samples. Nevertheless, compared with single chemotherapy, PD-L1 inhibitor with chemotherapy for SCLC patients presented beneficial OS (A et al., 2019) (40), and there is increasing evidence that shows that combination therapy, such as combined with chemotherapy, radiotherapy, and other targeted therapy, may overcome the low response to ICI among SCLC patients (38, 41, 42).

### PD-1 Inhibitors and irAEs in SCLC

Three PD-1 inhibitors, namely nivolumab, pembrolizumab, and tislelizumab, have been investigated in SCLC clinical trials as monotherapy or in combination chemotherapy.

In the nivolumab monotherapy arm of the Checkmate 032 study, the SCLC cohort comprised 109 chemotherapy-refractory SCLC patients who received nivolumab (3 mg/kg) as third-line or later-line therapy. After a median follow-up time of 28.3 months, the reported ORR was 11.9%. The median duration of response (DOR) was 17.9 months (range: 3.0–42.1). The median PFS and median OS were 1.4 months (95% CI: 1.3–1.6) and 5.6 months (95% CI: 3.1–6.8), respectively. Approximately 55% of patients in the nivolumab monotherapy group experienced treatment-related adverse events (TRAEs), and grade 3 to 4 TRAEs were reported in 11.9% of patients. Most irAEs reported in the study, including reactions of the skin (21.1%), endocrine system (9.2%), gastrointestinal tract (6.4%), hepatic system (4.6%), pulmonary system (1.8%), renal system (0.9%), and hypersensitivity/infusion reactions (3.7%), were mild (grade 1 to 2). Grade 3 to 4 irAEs included pneumonitis (1.8%), rash (0.9%), and aspartate aminotransferase (0.9%). One patient experienced grade 3–4 treatment-related encephalitis, and one death was reported due to treatment-related pneumonitis (43). Based on these promising results, the FDA approved nivolumab as third-line or later-line therapy for relapsed SCLC.

Nivolumab as second-line treatment for relapsed SCLC was evaluated in Checkmate 331 (NCT02481830). The preliminary results showed no significant differences in OS, PFS, ORR, and DOR during nivolumab monotherapy. The TRAEs were reported to be lower in the nivolumab group (grade 3 = 55%, grade 4 = 14%) than in the chemotherapy group (grade 3 = 90%, grade 4 = 73%) (35). Further details of TRAEs/irAEs associated with Checkmate 331 are still pending.

Pembrolizumab was another immunoglobulin G4 (IgG4) monoclonal antibody of PD-1 (44). Pembrolizumab first showed efficacy among the PD-L1-positive SCLC patients of the Keynote 028 study, with promising primary end point ORR (33.3%, 95% CI: 15.6–55.3%) and secondary end points (median DOR, median PFS, and median OS). The median DOR observed was 19.4 months, and the observed median PFS and median OS were 1.9 months and 9.7 months, respectively. TRAEs occurred in 66.7% of patients, and the most common events include arthralgia (16.6%), asthenia (16.6%), rash (16.7%), diarrhea (12.5%), and fatigue (12.5%). Two patients (8.3%) experienced treatment-related grade 3 to 5 AEs (one grade 3 bilirubin elevation and one grade 5 colitis/intestinal ischemia) (45).

Then, the Keynote 158 study enrolled 107 patients with SCLC (14% were PD-L1 positive) who received 200 mg of pembrolizumab treatment. Reportedly, the overall ORR was 18.7%. Significantly, difference was found in the ORR, median PFS, and median OS of the PD-L1-positive and PD-L1-negative cohorts. The ORR, median PFS, and median OS in the PD-L1-positive cohort presented superiority to that of the PD-L1-negative cohort. TRAEs were reported in 60% of all enrolled patients, with 12% of patients experiencing grade 3 to 4 TRAEs. Additionally, a total of 33% of patients experienced irAEs, with 5% of patients experiencing (suffering) grade 3-4 irAEs. The most common irAEs were hypothyroidism and hyperthyroidism, which occurred in 12% and 7% of the patients, respectively. Severe skin reactions were reported in 3% patients. Adrenal insufficiency, nephritis, pneumonitis and pancreatitis were all reported in 2% of the patients (46). In the according to subsequent pooled analysis of Keynote 028 and Keynote 158, the ORR was 19.3% (95% CI: 11.4–29.4) and the median time to response was 2.1 months (range: 1.7–4.1). The median PFS and median OS was 2.0 months (95% CI: 1.9–3.4) and 7.7 months (95% CI: 5.2–10.1), respectively. TRAEs occurred in 61.4% of the patients, with 7.2% of patients experiencing grade 3 TRAEs. IrAEs were considered to have occurred in 24.1% of patients, with the most common events being hypothyroidism, hyperthyroidism, and infusion reactions. Grade 3 irAEs occurred in 6.0% patients, including colitis, adrenal insufficiency, pancreatitis, and pneumonitis. Most of the irAEs could be alleviate by systemic corticosteroid treatment (47). Based on these promising results, pembrolizumab was also approved as third-line or later-line therapy for relapsed SCLC.

Regarding maintenance therapy, pembrolizumab monotherapy did not show any improvement in median PFS for patients with ES-SCLC (48). The median PFS of 45 enrolled patients was 1.4 months (95% CI: 1.3–2.8), and the median OS was 9.6 months (95% CI: 7.0–12). Higher median PFS and higher median OS were observed in patients with tumor stromal expressing PD-L1. Most reported TRAEs were mild (grade 2 or lower). Three adverse events, including rash (18%), hypothyroidism (9%), and type I diabetes mellitus with diabetic ketoacidosis (11%), were considered asirAEs.

Pembrolizumab was well tolerated during consolidation thoracic radiotherapy in a combination setting. In a single-institution phase I trial, after induction chemotherapy, 33 ES-SCLC patientwere treated with 45 Gy thoracic radiotherapy plus pembrolizumab. No dose-limiting toxicities were observed in the first 35 days, and the median PFS and OS were 6.1 months and 8.4 months, respectively. No grade 4 or 5 treatment-related toxicities were reported. TRAEs occurred in 6% of patients, but the investigator considered these were unlikely to be related to treatment (49). Furthermore, pembrolizumab combined with concurrent chemoradiation therapy were assessed in LS-SCLC patients, with the median follow-up time of 23.1 months, and the reported median PFS and median OS were 19.7 months and 39.5 months, respectively. Most TRAEs were mild; only one grade 4 respiratory failure and two grade 4 neutropenia were reported. Fatigue, dysphagia, dyspnea, and anemia were the most common grade 1-2 TRAEs. Conversely, neutropenia and anemia were the most common grade 3 TRAEs. Treatment-related pneumonitis was reported in 15% of patients (49).

In the NCT02551432 study, it was reported that the pembrolizumab plus paclitaxel as second-line therapy in relapsed or refractory SCLC patients were not inferior to the traditional second-line chemotherapy. The reported ORR was 23.1% (95% CI: 6.9–39.3%). The median PFS and median OS were 5.0 months (95% CI: 2.7–6.7) and 9.1 months (95% CI: 6.5–15.0), respectively. All enrolled patients experienced adverse events., with ~46% grade 3 or 4 adverse events were reported, including febrile neutropenia, neutropenia, asthenia, hyponatremia, and type I diabetes (50).

Recently, combination of pembrolizumab plus etoposide/platinum (EP) as the first-line therapeutic regimens for ES-SCLC patients were also evaluated in the Keynote 604 study. The results indicate that pembrolizumab plus EP significantly improved PFS, but the significance threshold for OS was not reached. A total of 24.7% irAEs were reported in the pembrolizumab plus chemotherapy group, and 10.3% of irAEs were reported in the chemotherapy group. The most common irAEs were hypothyroidism, hyperthyroidism, and pneumonitis. Grade 3 irAEs reported in the pembrolizumab plus chemotherapy group were 7.2% vs. the 0.9% in the chemotherapy group. The most common grade 3 irAEs were severe skin reactions (1.8%), pneumonitis (1.3%), and hepatitis (1.3%) (51).

Tislelizumab is, a PD-1 antibody with high affinity and specificity, was explored in the first-line SCLC treatment setting in a phase II study (NCT03432598). 17 Chinese ES-SCLC patients were treated with tislelizumab (200 mg) plus etoposide and platinum. The ORR was 77% (95% CI: 50.1–93.2). The median PFS and median OS in the SCLC cohort was 6.9 months and 15.6 months.IrAEs in the SCLC cohort included thyroid disorders (29.4%), pneumonitis (5.9%), and type 1 diabetes mellitus (5.9%) (52).

### PD-L1 Inhibitors and irAEs in SCLC

PD-L1 inhibitor atezolizumab and durvalumab as combination agents with platinum–etoposide indicated a promising profile as first-line therapy for ES-SCLC patients.

NCT01375842, a first phase Ia study, assess atezolizumab's single agent clinical activity in patients with ES-SCLC. The study enrolled 17 patients, and after 6.7 months of follow-up, the confirmed ORR was 6%. The reported median PFS and median OS were 1.5 months and 5.9 months, respectively. Most patients (65%) experienced various grades of TRAEs, with the most common being fatigue (24%). One grade 3 pneumonitis and one grade 5 hepatic failure were reported (53).

The IMpower133 trial further evaluated atezolizumab plus chemotherapy as first-line treatment option for ES-SCLC patients. The median OS and median PFS in the combined therapy group were both longer than in monotherapy group. The secondary end points ORR and DOR were similar between the two groups. IrAEs occurred in 39.9% of patients in the atezolizumab group and in 24.5% of patients in the placebo group. The most common irAEs included rash (18.7 vs. 10.2%), hypothyroidism (12.6 vs. 0.5%), hepatitis (7.1 vs. 4.6%), infusion-related reactions (5.6 vs. 5.1%), hyperthyroidism (5.6 vs. 2.6%), pneumonitis (2.0 vs. 2.6%), and colitis (1.5 vs. 0%). The grade 3-4 irAEs reported in the atezolizumab group included rash (2.0%), hepatitis (1.5%), infusion-related reaction (2.0%), pneumonitis (0.5%), colitis (1.0%), pancreatitis (0.5%), rhabdomyolysis (0.5%), nephritis (0.5%), and Guillain–Barre syndrome (0.5%) (54). A following study further presented the safety and tolo data of the induction and maintenance phases of the IMpower133 trial. The results were similar to those that had previously been reported. IrAEs were more frequently reported in the atezolizumab arm during both induction therapy and maintenance therapy, with rash and hypothyroidism being the most common irAEs (40).

In contrast, atezolizumab monotherapy failed to demonstrate clinic efficacy for relapsed SCLC patients in second line management setting in the non-comparative phase II IFCT-1603 study. The ORR of the atezolizumab group was only 2.3%, and the median PFS of the chemotherapy group was longer than that of the atezolizumab group. Difference in median OS was observed between the two groups. IrAEs in the atezolizumab group included hepatitis (4.2%), colitis (4.2%), arthralgia (6.3%), dysthyroidism (4.2%), musculoskeletal and connective tissue disorders (12.5%), and gastrointestinal disorders (18.8%). Most of the irAEs were mild (grade 1 or 2) (55).

In March 2020, another anti-PD-L1 antibody, durvalumab, in combination with platinum–etoposide was approved by the FDA as first-line therapy of ES-SCLC patients based on the findings of the CASPIAN trial (NCT03043872). The CASPIAN study was designed as an open-label, phase 3 trial to assess durvalumab with or without tremelimumab in combination with platinum–etoposide as first-line treatment for ES-SCLC patients. Firstly, the result of durvalumab plus platinum–etoposide group and the platinum–etoposide group have been published in the planned interim analysis. The reported median OS in the durvalumab plus platinum–etoposide group was slightly longer than the platinum–etoposide group (13.0 months vs. 10.3 months, respectively). The median PFS and 6-month PFS was similar between the two groups. The 12-month PFS rates and 18-month OS rates were higher in the durvalumab plus platinum–etoposide arm. The confirmed ORR in the two groups was 68% and 58%, respectively. Reported TRAEs were similar between the two groups (89 and 90%, respectively). The grade 3–4 TRAEs were similar between the chemotherapy group and in the durvalumab plus chemotherapy group (both are 62%). IrAEs were experienced by 20% of patients in the durvalumab plus chemotherapy group, with only 5% of patients experiencing grade 3 or 4 irAEs. Only 3% of patients in the chemotherapy group experienced irAEs and the occurrence rate of grade 3 or 4 irAEs was <1%. The most common irAEs were hypothyroid events (9 vs. 1%), hyperthyroid events (5 vs. 0%), pneumonitis (3 vs. 1%), hepatic events (3 vs. 0%), dermatitis/rash (2 vs. 1%), and diarrhea/colitis (2 vs. <1%). Thyroiditis and type 1 diabetes mellitus only occurred in the durvalumab plus chemotherapy group. Other rare irAEs included one patient in the durvalumab plus chemotherapy group who experienced adrenal insufficiency, and one patient in the durvalumab plus chemotherapy group who experienced grade 3 or 4 pancreatic events. Grade 3 or 4 irAEs in the durvalumab plus chemotherapy group included pneumonitis (1%), hepatic events (2%), diarrhea/colitis (<1%), type 1 diabetes mellitus (2%), and pancreatic events (<1%). Deaths due to irAEs occurred in <1% of patients in each group. One therapy-related hepatotoxicity caused death in the durvalumab plus chemotherapy group and one therapy-related pneumonitis caused death in the platinum–etoposide group (56).

Similarly, the durvalumab monotherapy failed for relapsed SCLC patients. The confirmed ORR was only 9.5% (95% CI: 1.2–30.4). The median PFS and OS were 1.5 months (95% CI: 0.9–1.8) and 4.8 months (95% CI: 1.3–10.4). The 12-month OS rate was 27.6% (95% CI: 10.2–48.4). A total of 33.3% of patients had TRAEs, all grade 1 or 2. Nausea, fatigue, and maculo-papular rash were the most commonly reported TRAEs. No TRAEs led to discontinuation or death (36).

### CTLA-4 Inhibitors and irAEs in SCLC

CA184-041 (NCT00527735) was the first phase II study exploring the clinical efficiency and tolerability of the CTLA-4 inhibitor ipilimumab in ES-SCLC patients. Chemotherapy-naïve ES-SCLC patients were randomized to receive paclitaxel/carboplatin with either placebo, concurrent ipilimumab (ipilimumab plus paclitaxel/carboplatin followed by maintenance treatment with ipilimumab), or phased ipilimumab (paclitaxel/carboplatin administered before ipilimumab, followed by maintenance treatment with ipilimumab). Prolonged immune-related PFS (irPFS) was only reported in the phased ipilimumab group. Nonsignificant improvement in PFS and OS was observed in both the ipilimumab groups. The grade 3/4 TRAEs were more common in the ipilimumab-containing arms. The most common irAEs were related to skin events (rash and pruritus), gastrointestinal events (diarrhea), and liver function (increases in alanine aminotransferase and aspartate aminotransferase), both of which occurred more frequently in both the concurrent ipilimumab and the phased ipilimumab groups. Most grade 3/4 irAEs could be managed well after follow-up or systemic corticosteroid treatment. Thus, phased ipilimumab demonstrated both efficacy and safety in previously untreated ES-SCLC patients in this clinical study (57).

In another phase II open-label study (NCT01331525), 42 chemotherapy-naïve ES-SCLC patients were treated with six cycles of carboplatin and etoposide plus ipilimumab. The study did not meet the primary endpoint (1-year PFS). The median PFS and median OS were 6.9 months and 17.0 months, respectively. In total, 69.2% of patients experienced serious irAEs (≥ grade 3), with the most frequent irAEs being diarrhea and skin rash. Serious ipilimumab-related neurological adverse events (grade 3 or higher) were reported in 7.6% of patients. Moreover, five deaths related to ipilimumab occurred; two of which were reported shortly after treatment (cardiac arrest, neutropenic sepsis) and three occurred 4–5 months after the last treatment (pneumonia, autoimmune encephalitis, and sepsis). This study additionally presented an association between improved outcomes and baseline autoimmunity of the therapy (58).

The phase III study CA184-156 (NCT01450761) investigated the efficacy and safety of ipilimumab plus etoposide and platinum for newly diagnosed ES-SCLC patients. The results showed that the addition of ipilimumab to chemotherapy did not present a survival benefit in ES-SCLC patients. The median OS and median PFS was found to be similar between the chemotherapy plus ipilimumab group and the chemotherapy plus placebo group. Patients receiving ipilimumab had more TRAEs, which required discontinuation of therapy (18 vs. 2%). Gastrointestinal and skin-related AEs were the most common irAEs (34 vs. 29%). The other irAEs that presented in more than 5% of patients were diarrhea (25 vs. 10%), rash (19 vs. 3%), pruritus (12 vs. 2%), colitis (6 vs. 1%), alopecia (5 vs. 7%), endocrine irAEs (10 vs. 2%), and peripheral sensory neuropathy (2 vs. 1%). Moreover, 76% of grade 2-4 irAEs were completely resolved. Neurologic events required more time (28.9 weeks) to resolve compared with other irAEs (59).

### Double Check Point Inhibitors and irAEs in SCLC

Different from PD-1/PD-L1, CTLA-4 activates T cells in the early stage. In theory, the combination of these two inhibitors would be more effective than either of them alone. Previously, the combination of double checkpoint inhibitors has shown survival benefits in some solid tumors, such as advanced melanoma and relapsed malignant pleural mesothelioma (60, 61).

Some clinical trials of ICI combinations in SCLC patients have also been reported (62). In the Checkmate 451 study, ES-SCLC patients were randomly receiving nivolumab (1 mg/kg) plus ipilimumab (3 mg/kg), nivolumab (240 mg) as a single agent, or placebo. Neither nivolumab alone or in combination with ipilimumab significantly improved OS compared with placebo, and 86% of patients receiving nivolumab plus ipilimumab experienced adverse events. Toxicity-induced discontinuation of therapy was reportedly higher in the combination group than in the nivolumab monotherapy and placebo groups (32% vs. 9% vs. 1%, respectively). Moreover, the deaths of seven patients that were related to the treatment were reported in the nivolumab plus ipilimumab group (63).

In the Checkmate 032 study, eligible SCLC patients (both limited- and extensive-stage) were treated with nivolumab monotherapy (3 mg/kg) or nivolumab combined ipilimumab (nivolumab 1 mg/kg plus ipilimumab 1 mg/kg, nivolumab 1 mg/kg plus ipilimumab 3 mg/kg, nivolumab 3 mg/kg plus ipilimumab 1 mg/kg). The results showed that nivolumab plus ipilimumab significantly improved ORR in SCLC patients. In addition, the median OS was improved in the combination group, but the median PFS of the nivolumab (3 mg/kg) plus ipilimumab (1 mg/kg) group was similar to that of the nivolumab monotherapy group. Adverse events, including all grades of adverse events, were also higher in the combination group. Increased lipase and diarrhea were the most commonly reported grade 3–4 TRAEs. The incidence of discontinued treatment due to TRAEs was higher in the nivolumab (1 mg/kg) plus ipilimumab (3 mg/kg) cohort, whereas that of discontinued treatment due to TRAEs was similar in the nivolumab monotherapy (3 mg/kg) cohort and the nivolumab (3 mg/kg) plus ipilimumab (1 mg/kg) cohort. Two treatment-related deaths were reported in the nivolumab (1 mg/kg) plus ipilimumab (3 mg/kg) cohort (including myasthenia gravis and worsening of renal failure), and one treatment-related pneumonitis caused death in the nivolumab (3 mg/kg) plus ipilimumab (1 mg/kg) cohort. The 1-year survival rate of patients with high TMBs was significantly higher (59b).

In a phase I dose-exploration and expansion study (NCT02261220), another double check point inhibitor group, durvalumab in combination with tremelimumab, demonstrated a promising clinical activity for ES-SCLC patients who received prior systematic therapy. The confirmed ORR was 13.3%, and the median DOR was 18.9 months (95% CI: 16.3–18.9). The disease control rate at 16 weeks was 20.0% (95% CI: 7.7–38.6). The median PFS and median OS were 1.8 months and 7.9 months, respectively. The 12-month OS was 41.7% (95% CI: 23.3–59.2). However, 67% of patients experienced TRAEs, with 23% experiencing grade 3/4 TRAEs. Fatigue (23%) and pruritus (23%) were the most common TRAEs (64).

In arm A of the BALTIC (NCT02937818) phase II study, the efficacy of durvalumab plus tremelimumab in platinum-refractory/resistant ES-SCLC patients was further tested. In this study, 25 patients were treated with durvalumab (1,500 mg) plus tremelimumab (75 mg) for up to 4 months, followed by durvalumab (1,500 mg) until progressive disease or discontinuation. The reported ORR was 9.5% (95% CI: 1.17–30.38); 23.8% of patients had stable disease and 4.8% of patients had an unconfirmed partial response. Grade 3 or higher TRAEs were experienced by 19% of patients; however, the updated information has not yet been published (65).

On December 2020, the updated results of CASPIAN trial published the data of durvalumab plus chemotherapy group and durvalumab plus tremelimumab plus chemotherapy group. Safety profiles of the durvalumab plus chemotherapy group and the chemotherapy group were consistent with previously reported. Immune-mediated adverse events were reported in patients in the durvalumab plus tremelimumab plus chemotherapy group, durvalumab plus chemotherapy group and chemotherapy group were 36, 20, and 3%, respectively. Usually reported irAEs were hypothyroid events, hyperthyroid events, diarrhea or colitis and dermatitis or rash. Grade 3 or 4 immune mediated adverse events occurred in 14% patients in the durvalumab plus tremelimumab plus chemotherapy group, 5% patients in the durvalumab plus chemotherapy group, and <1% patients in chemotherapy group. Deaths caused by irAEs occurred in 1% patients receiving durvalumab plus tremelimumab plus chemotherapy (enterocolitis, pneumonitis, pneumonitis and hepatitis), 1% patients receiving durvalumab plus platinum–etoposide (hepatotoxicity and interstitial lung disease) and <1% receiving platinum–etoposide (pneumonitis) (66).

## Discussion

For many years, few breakthroughs in SCLCs have been reported. Chemotherapy and radiotherapy were the only effective therapeutic methods for ES-SCLC patients. However, in recent years, immunotherapy has brought new hope for patients with SCLC. Some ICIs have improved chemotherapy's efficacy in ES-SCLC patients, but a comprehensive understanding of the mechanisms and preclinical rationale of immunotherapy in SCLC patients is still required. In this review, we summarized the available clinical trial data on ICIs for the treatment of SCLC. We are particularly concerned about IRAES, which are often overlooked by existing reviews.

A systematic collection of the efficacy and safety data of ICIs in the treatment of SCLC is performed in this review. Two reviewers independently searched current literature from the Cochrane Library, Clinical Trials, PubMed, and MEDLINE databases, using the following key words: “Small cell lung cancer,” “immune checkpoint inhibitor,” “nivolumab,” “pembrolizumab,” “atezolizumab,” “avelumab,” “durvalumab,” and “ipilimumab.” Clinical trials reporting both efficacy and safety data were included. A total of 23 studies covering 5 PD-1/PD-L1 inhibitors and 1 CTLA-4 antibody were included.

To intuitively compare the efficacy of ICIs and the occurrence of irAEs in SCLC, we summarized the results in Tables 2, 3. Overall, the efficacy of different mechanisms in ICIs also varied (Table 2). The anti-PD1 inhibitors nivolumab and pembrolizumab as third-line therapy presented the tolerable response for relapsed SCLC. Pembrolizumab combined with thoracic radiotherapy or concurrent chemoradiation therapy presented a good degree of tolerance in preliminary findings. Pembrolizumab combined with platinum–etoposide as the first-line therapy for ES-SCLC patients improved PFS, but the significance threshold for OS was not reached. In contrast, nivolumab monotherapy and pembrolizumab combined with chemotherapy were all not superior to chemotherapy as second-line therapy in recurrent SCLC. Another anti-PD1, tislelizumab plus platinum–etoposide, presented a higher ORR for Chinese ES-SCLC patients, but the result needs to be validated in further studies with large sample sizes. Anti-PD-L1 inhibitors atezolizumab and durvalumab both improved the survival benefits of chemotherapy for SCLC patients, but atezolizumab monotherapy or durvalumab monotherapy failed in second-line therapy for refractory SCLC patients. The results of CTLA-4 inhibitors were also dismal. Ipilimumab monotherapy or combined with chemotherapy did not exhibit significant efficacy for newly diagnosed ES-SCLC patients and refractory ES-SCLC patients. Moreover, existing studies could not affirm the efficacy of the combined checkpoint inhibitors in SCLC, as the results of the durvalumab and tremelimumab arm of the CASPIAN study are still pending. However, the double checkpoint inhibitors increased the risk of irAEs. The overall irAEs' occurrence rate in patients with SCLC ranged from 20% (CASPIAN) to 57% (NCT01450761) (Table 3). The most commonly reported irAEs were rash, diarrhea, hypothyroidism/hyperthyroidism, colitis, and pneumonia. In addition, nephritis, hepatitis, pancreatitis, and some nervous system-related irAEs were observed. The rate of high grade (grade ≥3) irAEs was less than 10% in most trials, and most irAEs were manageable through systematic therapy in most studies. Pneumonitis was the most frequently reported death-related irAE. Hypothyroidism and hyperthyroidism are reported relatively less frequently in CTLA-4 inhibitors compared with PD-1/PD-L1 inhibitors, which was consistent with the findings of a previous study (67). No special safety data were reported. To determine different rates and types of irAEs in SCLC, we compared irAEs reported in other cancer type cohorts from the Checkmate 032, Keynote 028, Keynote 158, NCT01375842 and NCT03432598(Supplementary Table 1) studies; however, no specific irAEs of SCLC were found. Furthermore, it is difficult to further quantify and compare these indicators because of the variations between studies in terms of the length of median follow-up. Moreover, irAEs in most trials were evaluated by the investigators, which might not be objective and could be lacking a uniform standard. Some studies only reported TRAEs instead of irAEs, and the details of irAEs in most trials are unavailable.

**Table 2:** Clinical trials' efficacy data of ICIs in patients with SCLC.

**Agent**	**Trial**	**Phase**	**Line of therapy**	**Population**	**Treatment arms**	**Primary end point**	**Secondary end points**	**Median follow-up time**	**Publish year**
Nivolumab	CheckMate032 (NCT01928394)	Phase I/II	Third or later line	SCLC	NIvolumab 3 mg/kg	ORR: 11.9% (95% CI: 6.5–19.5)	mDOR: 17.9 m (95% CI:3.0–42.1); mOS: 5.6 m (95% CI: 3.1–6.8); mPFS: 1.4 m (95% CI: 1.3–1.6)	28.3 m	2018
	CheckMate331 (NCT02481830)	Phaseiii	Second line	Relapsed SCLC	Nivolumab 240 mg	mOS: 7.46 m (95% CI: 5.65–9.20)	mPFS: 1.45 m (95% CI:1.41- 1.51); ORR: 13.7% (95% CI:10.0–18.3); DOR:72%	15.8 m	2018
Pembrolizumab	KEYNOTE028 (NCT02054806)	Phase Ib	Third line	ES-SCLC	Pembrolizumab 10 mg/kg	ORR: 33% (95 CI: 16–55%)	mDOR: 19.4 m (95% CI:3.6–20.0); mPFS:1.9 m (95% CI:1.7–5.9); mOS: 9.7 m (95% CI: 4.1- not reached).	9.8 m	2017
	KEYNOTE158 (NCT02628067)	Phase II	Third line	ES-SCLC	Pembrolizumab 200 mg	ORR: 18.7% (95% CI: 11.8% −27.4%)	mPFS: 2.0 m (95%CI: 1.9–2.1); mOS: 8.7 m (95% CI: 5.6–12)	10.1 m	2018
	pool analysis of KEYNOTE028 and KEYNOTE158	Phase Ib/phase II	Third line	ES-SCLC	Pembrolizumab 10 mg/kg or 200 mg	ORR: 19.3% (95% CI: 11.4–29.4%)	mPFS: 2.0 m (95% CI: 1.9–3.4); mOS:7.7 m (95% CI: 5.2–10.1)	25.9 m	2020
	Gadgeel et al. (48)	Phase II	Maintenance therapy	ES-SCLC	Pembrolizumab 200 mg	mPFS: 1.4 m (95% CI: 1.3–2.8)	mOS: 9.6 m (95% CI: 7.0–12)	5 w	2018
	NCT02402920	Phase I	Second line	ES-SCLC	45 Gy thoracic radiotherapy +pembrolizumab 50–200 mg	Safety	mPFS: 6.1 m (95% CI 4.1–8); mOS: 8.4 m (95%; CI: 6.7–10.1)	7.3 m	2020
	Welsh et al. (49)	phase I/II	-	LS-SCLC	Concurrent chemoradiotherapy +pembrolizumab 100–200 mg	Safety	mPFS:19.7 m (95% CI 8.8–30.5); mOS:39.5 months (95% CI:8.0–71.0)	23.1 m	2020
	NCT02551432	Phase II	Second line	ES-SCLC	Paclitaxel +pembrolizumab 200 mg	ORR: 23.1% (95% CI: 6.9–39.3)	mPFS: 5.0 m (95% CI: 2.7–6.7); mOS:9.1m (95% CI: 6.5–15.0)	11.1 m	2019
	KEYNOTE604 (NCT03066778)	Phase III	First line	ES-SCLC	Pembrolizumab 200 mg + etoposide+platinum	mPFS: 4.5 m (95% CI: 4.3–5.4); mOS: 10.8 m (95% CI: 9.2–12.9)	ORR: 70.6% (95% CI: 64.2–76.4); mDOR: 4.2 m (95% CI:1.01–26.01)	22 m	2020
					Placebo + etoposide +platinum	mPFS: 4.3 m (95% CI: 4.2–4.4); mOS: 9.7 m (95% CI: 8.6–10.7)	ORR: 61.8% (95% CI: 55.1–68.2); mDOR: 3.7 m (95% CI:1.41–25.81)		
Tislelizumab	NCT03432598	Phase II	First line	ES-SCLC	Tislelizumab 200 mg + etoposide+platinum	ORR: 77% (95% CI: 50.1–93.2)	mPFS: 6.9 m (95% CI: 4.9–10.09)	15.3 m	2020
Atezolizumab	NCT01375842	Phase Ia	First line	ES-SCLC	Atezolizumab 15 mg/kg or 1200 mg	Safety	ORR: 6%; mPFS: 1.5 m (95% CI: 1.2–2.7); mOS: 5.9 m (95% CI: 4.3–20.1)	6.7 m	2016
	IMpower133 (NCT02763579)	Phase I/III	First line	ES-SCLC	Atezolizumab 1,200 mg+carboplatin + etoposide	mOS: 12.3 m (95% CI:10.8–15.9); mPFS:5.2 m (95% CI: 4.4–5.6)	ORR: 60.2% (95% CI:53.1–67.0); DOR: 4.2 m (95%CI: 1.4+ −19.5)	13.9 m	2018
					Placebo+ carboplatin+ etoposide	mOS:10.3 m (95% CI: 9.3–11.3); mPFS: 4.3 m (95% CI: 4.2–4.5)	ORR: 64.4% (95% CI: 57.3–71.0); DOR: 3.9 m (95% CI:2.0–16.1+)		
	IFCT-1603 (NCT03059667)	Phase II	Second line	relapsed ES-SCLC	Atezolizumab 1,200 mg	ORR: 2.3% (95% CI: 0.0–6.8)	mPFS:1.4 m (95%CI: 1.2–1.5); mOS: 9.5 m (95% CI: 3.2–14.4)	13.7 m	2019
					Chemotherapy	ORR: 10% (95% CI: 0.0–23.1)	mPFS:4.3 m (95%CI: 1.5–5.9); mOS:8.7m (95% CI:4.1–12.7)		
Durvalumab	CASPIAN (NCT03043872)	Phase III	First line	ES-SCLC	Durvalumab 1,500 mg + etoposide+ platinum	mOS: 13.0 m (95% CI: 11.5–14.8)	mPFS: 5.1 m(95% CI 4.7–6.2); ORR: 68%	14.2 m	2019
					Etoposide+platinum	mOS: 10.3 m (95%CI: 9.3–11.2)	mPFS: 5.4 m (95% CI:4.8–6.2); ORR: 58%		
	Goldman et al. (36)	Phase I/II	Second line	Relapsed SCLC	Durvalumab 10 mg/kg	Safety	ORR: 9.5% (95% CI: 1.2–30.4); mPFS: 1.5 m (95% CI: 0.9–1.8); mOS: 4.8 m (95% CI: 1.3–10.4)	NA	2018
Ipilimumab	CA184-041 (NCT00527735)	Phase II	First line	ES-SCLC	Placebo/ paclitaxel /carboplatin	irPFS: 5.3 m	mOS: 9.9 m; irBORR: 53% (95% CI: 38–68%); irDCR: 96% (95% CI: 85–100%)	11.1 m	2013
					Ipilimumab 10 mg/kg/placebo+ paclitaxel/ carboplatin(concurrent)	irPFS: 5.7 m	mOS: 9.1 m; irBORR: 49% (95% CI: 33–65%); irDCR: 81% (95% CI: 67–92%)		
					Ipilimumab 10 mg/kg/placebo+ paclitaxel/ carboplatin(phased)	irPFS: 6.4 m	mOS: 12.9 m; irBORR: 71% (95% CI: 55%- 84%); irDCR: 93% (95% CI: 81–99%)		
	NCT01331525	Phase II	First line	ES-SCLC	Ipilimumab 10 mg/kg+ carboplatin+ etoposide	not meet	mPFS: 6.9 m (95%CI: 5.5–7.9); mOS: 17.0 m (95% CI: 7.9–24.3); median irPFS:7.3 m (95% CI: 5.5–8.8)	8.5 m	2016
	NCT01450761	Phase III	First line	ES-SCLC	Ipilimumab 10 mg/kg+etoposide +platinum (cisplatin+ carboplatin)	mOS: 11.0 m	mPFS: 4.6 m; mDOR: 4.01 (95% CI: 3.32–4.17)	10.5 m	2016
					Placebo+ etoposide+ platinum (cisplatin+ carboplatin)	mOS: 10.9 m	mPFS: 4.4 m; mDOR: 3.45 m (95% CI: 3.25–4.07)	10.2 m	
Nivolumab +ipilimumab	CheckMate451 (NCT02538666)	Phase III	Maintenance therapy	Relapsed ES-SCLC	Nivolumab 1 mg/kg + ipilimumab 3 mg/kg	mOS: 9.17 m (95% CI:8.15–10.25)	mPFS: 1.74 (95% CI: 1.48–2.63)	9 m	2019
					Nivolumab 1 mg/kg	mOS: 10.41 m (95% CI:9.46–12.12)	mPFS: 1.87 (95% CI: 1.61–2.63)		
					Placebo	mOS: 9.56 m (95% CI:8.18–11.01)	mPFS: 1.45 (95% CI: 1.41–1.48)		
	CheckMate032 (NCT01928394)	Phase I/II	Second or later line	SCLC	Nivolumab 3mg/kg	ORR:10%	mOS: 4.4 m (95% CI: 3.0–9.3); mPFS: 1.4 m (95% CI: 1.4–1.9)	198.5 d	2016
					Nivolumab 1 mg/kg + ipilimumab 3 mg/kg	ORR:23%	mOS: 7.7 m (95% CI: 3.6–18.0); mPFS: 2.6 m (95% CI: 1.4–4.1)	361.0 d	
					Nivolumab 3 mg/kg + ipilimumab 1 mg/kg	ORR:19%	mOS: 6.0 m (95% CI: 3.6–11.0); mPFS: 1.4 m (95% CI: 1.3–2.2)	260.5 d	
Durvalumab+ tremelimumab	NCT02261220	Phase I	Third line	ES-SCLC	Durvalumab 20 mg/kg+tremelimumab 1 mg/kg	safety	ORR: 13.3%; DOR: 18.9 m (95% CI: 16.3–18.9); mPFS: 1.8 m (95% CI: 1.0–1.9); mOS: 7.9 m (95% CI: 3.2–15.8)	NR	2018
	BALTIC (NCT02937818)	Phase II	First line	ES-SCLC	Durvalumab 1,500 mg + tremelimumab 75 mg	ORR: 9.5% (95% CI: 1.17–30.38)	12 weeks DCR: 38.1%	14 w	2018
Durvalumab+ tremelimumab	CASPIAN (NCT03043872)	Phase III	First line	ES-SCLC	durvalumab 1,500 mg + tremelimumab 75 mg+platinum +etoposide	mOS: 10.4 m (95% CI 9.6–12.0)	mPFS: 4.9 m (95% CI 4.7–5.9); unconfirmed objective response: 74%	25.1 m	2021
					Durvalumab 1,500 mg+platinum +etoposide	mOS: 12.9 m (95% CI: 11.3–14.7)	mPFS: 5.1 m (95% CI 4.7–6.2); unconfirmed objective response: 79%		
					Platinum+ etoposide	mOS: 10.5 m (95%CI: 9.3–11.2)	mPFS: 5.4 m (95% CI 4.8–6.2); unconfirmed objective response: 71%		

*mDOR, median disease control rate; mOS, median overall survival; mPFS, median progression-free survival; ORR, overall response rate; irPFS, immune-related progression-free survival; irBORR, immune related best overall response rate; irDCR, immune related disease control rate; +, denotes a censored observation; NA, not available*.

**Table 3:** Clinical trials' safety data of ICIs in patients with SCLC.

**Target**	**NCT number**	**Treatment**	**Enrolled number**	**TRAEs**	**TRAEs (grade≥3)**	**IRAEs**	**IRAEs (grade≥3)**	**Most common TRAEs/IRAEs**	**TRAEs/IRAEs (grade≥3)**	**Death related to TRAEs/IRAEs**
PD-1	CheckMate032 (NCT01928394)	Nivolumab 3 mg/kg	109	55%	11.9%	48%	4%	• **IRAEs:** • Skin reactions (21.1%), • Endocrine (9.2%), • Gastrointestinal (6.4%), • Hepatic (4.6%), • Infusion reaction (3.7%), • Pulmonary (1.8%), renal (0.9%)	• **IRAEs:** • Pneumonitis (1.8%), • Rash (0.9%), • AST increased (0.9%)	Pneumonitis (0.9%)
	CheckMate331 (NCT02481830)	Nivolumab 240 mg	282	55%	14%	NA	NA	NA	NA	NA
		Chemotherapy (either topotecan or amrubicin)	265	90%	73%	NA	NA	NA	NA	NA
	KEYNOTE028 (NCT02054806)	Pembrolizumab 10 mg/kg	24	66.7%	8.3%	NA	NA	• **TRAEs:** • Arthralgia (16.6%), • Asthenia (16.6%), • Rash (16.7%), • Diarrhea (12.5%), fatigue (12.5%)	• **TRAEs:** • Grade 3 bilirubin elevation (4.2%), • Grade 5 colitis/intestinal ischemia (4.2%)	Colitis and intestinal ischemia (4.2%)
	KEYNOTE158 (NCT02628067)	Pembrolizumab 200 mg	107	60%	12%	33%	5%	• **IRAEs:** • Hypothyroidism (12%), • Hyperthyroidism (7%), • Severe skin reactions (3%), • Adrenal insufficiency (2%), nephritis (2%), • Pneumonitis (2%), pancreatitis (2%)	• **IRAEs:** • Severe skin reactions (1%), • Adrenal insufficiency (1%), • Pancreatitis (2%), • Pneumonitis (1%), colitis (1%)	• Pneumonitis (0.9%), • Encephalopathy (0.9%)
	Pool analysis of KEYNOTE-028 and KEYNOTE-158	Pembrolizumab 10 mg/kg or 200 mg	83	61.4%	7.2%	24.1%	6%	• **IRAEs:** • Hypothyroidism (10.8%), • Hyperthyroidism (6.0%), • Infusion reactions (3.6%), • Colitis (2.4%), • Severe skin reactions (1.2%), • Adrenal insufficiency (1.2%), • Pneumonitis (1.2%), • Nephritis (1.2%), • Thyroiditis (2.4%), • Pancreatitis (1.2%), hepatitis (1.2%)	• **IRAEs:** • Colitis (2.4%), • Adrenal insufficiency (1.2%), • Pancreatitis (1.2%), • Pneumonitis (1.2%)	• Pneumonia (1.2%), • Intestinal ischemia (1.2%), • Encephalopathy (1.2%)
	Gadgeel et al. (48)	Pembrolizumab 200 mg	45	NA	NA	NA	NA	• **IRAEs:** • Rash (18%), • Hypothyroidism (9%), • Type I diabetes mellitus with diabetic Ketoacidosis (11%)	None	None
	NCT02402920	45 Gy thoracic radiotherapy + pembrolizumab 50–200 mg	33	NA	NA	NA	NA	NA	NA	NA
	Welsh et al. (49)	Concurrent chemoradiotherapy + pembrolizumab 100–200 mg	40	100%	88%	NA	NA	• **TRAEs:** • Fatigue (60%), • Dysphagia (58%), • Dyspnea (50%), • Esophagitis (43%), • Nausea (35%)	• **TRAEs:** • Anemia (13%), • Neutropenia (13%), • Lung infection (8%), • Pneumonitis (8%)	None
	NCT02551432	Paclitaxel + pembrolizumab 200 mg	26	100%	46%	NA	NA	• **TRAEs:** • Peripheral sensory neuropathy (57.7%), • Myalgia (34.6%), • Anemia (23.1%), • Diarrhea (23.1%), • Anorexia (19.2%), • Pneumonia (19.2%)	• **TRAEs:** • Neutropenia (7.7%), • Febrile neutropenia (7.7%), • Asthenia (7.7%), • Hyponatremia (7.7%), • Type I diabetes mellitus (3.9%), • Anemia (3.9%), • Myalgia (3.9%)	None
	KEYNOTE-604 (NCT03066778)	Pembrolizumab 200 mg + etoposide + platinum	223	97.8%	63.7%	24.7%	8.1%	• **IRAEs:** • Hypothyroidism (10.3%), • Hyperthyroidism (6.7%), • Pneumonitis (4.0%), • Severe skin reactions (2.2%), • Hepatitis (1.8%), • Colitis (1.3%), • Adrenal insufficiency (0.9%), • Hypophysitis (0.9%), • Nephritis (0.9%), • Encephalitis (0.9%), • Myositis (0.4%), • Pancreatitis (0.4%), • Type 1 diabetes mellitus (0.4%), • Uveitis (0.4%)	• **IRAEs:** • Severe skin reactions (1.8%), pneumonitis (1.3%), • Hepatitis (1.3%), • Adrenal insufficiency (0.9%), • Hyperthyroidism (0.4%), • Colitis (0.4%), • Nephritis (0.4%), • Myositis (0.4%), • Pancreatitis (0.4%), • Type 1 diabetes mellitus (0.4%), uveitis (0.4%)	• Neutropenic sepsis (1.3%), • Cardiopulmonary failure (0.4%), • Respiratory failure (0.4%), • Sepsis (0.4%)
		Placebo + etoposide platinum	223	95.5%	61%	10.3%	0.9%	• **IRAEs:** • Hypothyroidism (2.2%), • Hyperthyroidism (2.7%), • Pneumonitis (2.2%), • Severe skin reactions (0.9%), • Colitis (0.9%), • Adrenal insufficiency (0.4%), • Myasthenic syndrome (0.4%), • Myocarditis (0.4%)	• **IRAEs:** • Colitis (0.9%)	Neutropenic sepsis (0.4%)
	NCT03432598	Tislelizumab 200 mg + etoposide + platinum	17	100%	76.5%	35.3%	None	• **IRAEs:** • Thyroid disorders (29.4%), • Pneumonitis (5.9%), • Type 1 diabetes mellitus (5.9%)	None	None
PD-L1	NCT01375842	Atezolizumab 15 mg/kg or 1,200 mg	17	65%	17.6%	NA	NA	• **TRAEs:** • Fatigue (24%)	• **TRAEs:** • Pneumonitis (5.9%), • Hepatic failure (5.9%)	None
	IMpower133 (NCT02763579)	Atezolizumab 1,200 mg + carboplatin + etoposide	198	94.9%	58.1%	39.9%	10.5%	• **IRAEs:** • Rash (18.7%), • Hypothyroidism (12.6%), • Hepatitis (7.1%), • Infusion-related reaction (5.6%), • Hyperthyroidism (5.6%), • Pneumonitis (2.0%), • Colitis (1.5%), • Pancreatitis (0.5%), • Severe cutaneous reaction (1.0%), • Rhabdomyolysis (1.0%), • Nephritis (0.5%), • Hypophysitis (0.5%), • Diabetes mellitus (0.5%), • Guillain–Barre Syndrome (0.5%)	• **IRAEs:** • Rash (2.0%), • Hepatitis (1.5%), • Infusion-related reaction (2.0%), • Pneumonitis (0.5%), • Colitis (1.0%), • Pancreatitis (0.5%), • Rhabdomyolysis (0.5%), • Nephritis (0.5%), • Guillain–Barre Syndrome (0.5%)	• Neutropenia (0.5%), • Pneumonia (0.5%), • Unspecified cause (0.5%)
		Placebo + carboplatin + etoposide	196	92.3%	57.6%	24.5%	2.5%	• **IRAEs:** • Rash (10.2%), • Hypothyroidism (0.5%), • Hepatitis (4.6%), • Infusion-related reaction (5.1%), • Hyperthyroidism (2.6%), • Pneumonitis (2.6%), • Pancreatitis (1.0%), • Adrenal insufficiency (1.0%), • Nephritis (0.5%), • Vasculitis (0.5%)	• **IRAEs:** • Infusion-related reaction (0.5%), • Pneumonitis (1.0%), pancreatitis(1.0%)	• Pneumonia (0.5%), • Septic shock (0.5%), • Cardiopulmonary failure (0.5%)
	IFCT-1603 (NCT03059667)	Atezolizumab 1,200 mg	48	NA	NA	22.9%	NA	• **IRAEs:** • Hepatitis (4.2%), • Colitis (4.2%), • Arthralgia (6.3%), • Dysthyroidism (4.2%)	NA	None
		Chemotherapy	24	NA	NA	NA	NA	NA	NA	None
	CASPIAN (NCT03043872)	Durvalumab 1,500 mg + etoposide + platinum	265	89%	46%	20%	5%	**IRAEs:** • Hypothyroid (9%), • Hyperthyroid (5%), • Pneumonitis (3%), • Hepatic events (3%), • Dermatitis/rash (2%), • Diarrhoea/colitis (2%)	• **IRAEs:** • Pneumonitis (1%), • Hepatic events (2%), • Diarrhoea/colitis (<1%), • Type 1 diabetes mellitus (2%), • Pancreatic (<1%)	• **IRAEs:** • Cardiac arrest (<1%), • Dehydration (<1%), • Hepatotoxicity (<1%), • Pancytopenia (<1%), sepsis (<1%)
		Etoposide + platinum	266	90%	52%	3%	<1%	• IRAEs: • Hypothyroid (1%), • Pneumonitis (1%), • Dermatitis/rash (1%), • Diarrhoea/colitis (<1%)	• **IRAEs:** • Pneumonitis (<1%)	• Pancytopenia (<1%), • Thrombocytopenia/ haemorrhage (<1%)
	Goldman et al. (36)	Durvalumab 10 mg/kg	21	33%	0%	NA	NA	• **TRAEs:** • Nausea (9.5%), • Fatigue (9.5%), • Rash maculo-papular (9.5%)	None	None
CTLA-4	CA184-041 (NCT00527735)	Placebo/paclitaxel/ carboplatin	44	91%	30%	NA	9%	• **TRAEs:** • Rash (4.5%), • Pruritus (11.4%), • Diarrhea (25%)	• **TRAEs:** • Diarrhea (11.3%)	None
		Ipilimumab 10 mg/kg/placebo + paclitaxel/carboplatin (concurrent)	42	84%	43%	NA	21%	• **TRAEs:** • rash (73.8%), • pruritus (57.1%), • diarrhean (50%)	• **TRAEs:** • Diarrhea (9.5%), • ALT increases (16.7%), • AST increase (11.9%), • Hepatitis (2%)	Hepatotoxicity (2.4%)
		Ipilimumab 10 mg/kg/placebo + paclitaxel/carboplatin (phased)	42	95%	50%	NA	17%	• **TRAEs:** • Rash (57.1%), • Pruritus (40.4%), • Diarrhea (57.1%)	• **TRAEs:** • Diarrhean(23.8%), • Colitis (2.38%), • Arthralgia (9.52%), • ALT increases (4.76%), • AST increases (7.14%), • Hepatitis (2%)	None
	NCT01331525	Ipilimumab 10 mg/kg + carboplatin + etoposide	39	100%	89.7%	NA	NA	• **IRAEs:** • Diarrhea (72%), • Skin rash (51%)	• **IRAEs:** • Ipilimumab related neurological adverse events (7.6%)	• Cardiac arrest (2.56%), • Neutropenic sepsis (2.56%), • Pneumonia (2.56%), • Autoimmune encephalitis (2.56%), • Sepsis (2.56%)
	NCT01450761	Ipilimumab 10 mg/kg + etoposide + platinum (cisplatin + carboplatin)	478	82%	48%	57%	20%	• **IRAEs:** • Diarrhea (25%), • Rash (19%), • Pruritus (12%), • Colitis (6%), alopecia (5%)	• **IRAEs:** • Rash (2%), • Pruritus (1%), • Diarrhea (7%), • Colitis (4%), • ALT increased (1%), • AST increased (1%)	•Colitis (0.42%), • Liver toxicity (0.21%)
		Placebo + etoposide + platinum (cisplatin + carboplatin)	476	76%	44%	28%	2%	•**IRAEs:** • Diarrhea (10%), • Rash (3%), • Pruritus (2%), • Colitis (1%), • Alopecia (7%)	• **IRAEs:** • Diarrhea (1%)	• Sepsis (0.21%), • Bone marrow suppression (0.21%)
Double ICIs	CheckMate 451 (NCT02538666)	Nivolumab 1 mg/kg + ipilimumab 3 mg/kg	278	86%	52%	NA	NA	NA	NA	NA
		Nivolumab 1 mg/kg	279	61%	12%	NA	NA	NA	NA	NA
		Placebo	273	50%	8%	NA	NA	NA	NA	NA
	CheckMate032	(NCT01928394)	Nivolumab 3 mg/kg	98	53%	13%	NA	NA	• **TRAEs:** • Fatigue (11%), • Pruritus (11%), • Diarrhoea (7%), • Nausea (7%), • Decreased appetite (6%), • Pneumonitis (3%), • Vomiting (3%), • Hypothyroidism (3%), • Hyperthyroidism (2%), • Rash (2%) All <1%	None
		Nivolumab 1 mg/kg + ipilimumab 3 mg/kg	61	79%	30%	NA	NA	• **TRAEs:** • Fatigue (26%), • Pruritus (20%), • Diarrhoea (21%), • Nausea (12%), • Decreased appetite (7%), • Pneumonitis (3%), • Vomiting (5%), • Hypothyroidism (17%), • Hyperthyroidism (11%), • Rash (19%)	• **TRAEs:** • Increased lipase (9%), • Diarrhoea (5%)	• Myasthenia gravis (2%), • Worsening of renal failure (2%)
		Nivolumab 3 mg/kg + ipilimumab 1 mg/kg	54	75%	19%	NA	NA	• **TRAEs:** • Fatigue (22%), • Pruritus (9%), • Diarrhoea (17%), • Nausea (7%), • Decreased appetite (11%), • Pneumonitis (6%), • Vomiting (9%), • Hypothyroidism (7%), • Hyperthyroidism (6%), • Rash (7%)	• **TRAEs:** • Dyspnoea (4%)	Pneumonitis (1%)
	NCT02261220	Durvalumab 20 mg/kg + tremelimumab 1 mg/kg	30	67%	23%	NA	NA	• **TRAEs:** Fatigue (23%), Pruritus (23%)	NA	NA
	BALTIC (NCT02937818)	Durvalumab 1,500 mg + tremelimumab 75 mg	25	NA	19%	NA	NA	NA	NA	NA
	CASPIAN (NCT03043872)	Durvalumab 1,500 mg + tremelimumab 75 mg + platinum + etoposide	266	90%	55%	36%	14%	• **IRAEs:** • Hypothyroid events (9%), • Hyperthyroid events (8%), • Diarrhoea/colitis (8%), • Dermatitis/rash (7%), • Hepatic events (4%), • Pneumonitis (3%)	• **IRAEs:** • Diarrhoea/colitis (3%), • Dermatitis/rash (2%), • Hepatic events (3%)	• Enterocolitis (0.5%), • Pneumonitis (0.5%), • Pneumonitis and hepatitis in the same patient (0.5%)
		Durvalumab 1,500 mg + platinum + etoposide	265	89%	46%	20%	5%	• **IRAEs:** • Hypothyroid events (9%), • Hyperthyroid events (5%), • Hepatic events (3%), • Pneumonitis (3%)	• **IRAEs:** • Diarrhoea/colitis (1%), • Type 1 diabetes mellitus (2%)	• Hepatotoxicity (0.5%), • Interstitial lung disease (0.5%)
		Platinum + etoposide	266	90%	52%	3%	<1%	• **IRAEs:** • Hypothyroid events (1%), • Diarrhoea/colitis (1%), • Pneumonitis (1%)	• **IRAEs:** • Pneumonitis (<1%)	Pneumonitis (<1%)

*AST, aspartate aminotransferase; ALT, alanine transaminase; NA, not available*.

In the 23 trials included (Table 2) in this review, the most commonly reported irAEs/TRAEs were mild. Pneumonitis was the most frequently reported death-related irAE. Other death-related irAEs include colitis/intestinal ischemia, encephalopathy, neutropenic sepsis, cardiopulmonary failure, hepatotoxicity, myasthenia gravis, worsening of renal failure, sepsis, and septic shock. Nevertheless, these only account a tiny proportion of irAEs, usually less than 5%. Serious irAEs were the indicator for ICI reduction or discontinuation in most studies, but the treatment details of irAEs were not described.

Some guidelines have been published for the diagnosis and management of irAEs (68, 69). IrAEs are graded according to the Common Terminology Criteria for Adverse Events. Mild irAEs graded 1 or 2 could gradually disappear after the discontinuation of ICIs. Moreover, the early identification is of great importance for the management of irAEs. Serious irAEs (grade ≥3) threaten the patients' life, corticosteroid therapy was usually needed, and the associated complications, such as infection, were also a source of concern. During the period of clinical therapy, the irAEs had greater complexity, hence individualized treatment and management strategies could be a future research direction.

Furthermore, the study areas of irAEs in SCLC patients that should be addressed are as follows: [1] peculiar irAEs, such as Fanconi syndrome, which was reported in an ES-SCLC patient after he received nivolumab plus ipilimumab as second-line therapy (70); [2] the occurrence rate of irAEs, as this was higher in a real-world report (71); [3] the difference in irAEs between SCLC and NSCLC, as a previous meta-analysis has reported that the occurrence of ICI-related TRAEs in SCLC patients was higher than that in NSCLC patients (72); [4] irAEs of other ICI agents and combination therapies, as new target ICI agents and combination strategies are emerging in SCLC (73); and [5] irAEs of specific populations, as patients with autoimmune diseases are usually excluded from clinical trials, but many patients with SCLC experience paraneoplastic syndromes, and therefore the advantages or disadvantages of ICIs for these populations should be explored in future studies.

## Conclusion and Prospects

The current review summarizes the efficacy and safety data of ICIs in all existing clinical trials in the SCLC treatment field. ICI agents generally demonstrate a promising clinical activity in SCLC therapy, with manageable irAEs, although more detailed data are required. Future study directions include finding reliable biomarkers for the selection of patients that will most benefit from therapy, and verifying the rationale of various combination therapeutic regimens. Moreover, further details regarding irAEs are encouraged to be record and reported in future investigations, which could be of great significance for clinical practice and would benefit the increasing number of patients with SCLC.

## Author Contributions

WH, XZ, CY, and HZ: conception and design and collection and assembly of data. CY: administrative support. CY and HZ: provision of study materials or patients. WH and XZ: manuscript writing. All authors final approval of manuscript.

## Conflict of Interest

The authors declare that the research was conducted in the absence of any commercial or financial relationships that could be construed as a potential conflict of interest.
