# Supplementary material for: Immune Check Point Inhibitors and Immune-Related Adverse Events in Small Cell Lung Cancer
Source: Front Oncol. 2021 Mar 18;11:604227. doi: 10.3389/fonc.2021.604227 (PMC8016392; doi:10.3389/fonc.2021.604227)
Supplement: Supplementary file 1 [file Table_1.docx]

**Supplement Table 1. IrAEs reported in other cancer type cohorts from the Checkmate 032, Keynote 028, Keynote 158, NCT01375842 and NCT03432598.**

| Target | NCT number | Treatment | Tumor type | Enrolled number | TRAEs | TRAEs (grade≥3) | IRAEs | IRAEs (grade≥3) | Most common TRAEs/IRAEs | Most commonTRAEs/IRAEs (grade≥3) | Death related to TRAEs/IRAEs | reference |
| --- | --- | --- | --- | --- | --- | --- | --- | --- | --- | --- | --- | --- |
| PD-1 | CheckMate032 (NCT01928394) | nivolumab 3 mg/kg | small cell lung cancer | 109 | 55.0% | 11.9% | 47.7% | 3.6% | **IRAEs:** skin reactions (21.1%),  endocrine (9.2%),  gastrointestinal (6.4%),  hepatic (4.6%),  infusion reaction (3.7%),  pulmonary (1.8%),  renal (0.9%) | **IRAEs:** pneumonitis (1.8%),  rash (0.9%),  AST increased (0.9%) | pneumonitis (0.9%) | Ready et al., 2019 |
|  |  |  | metastatic urothelial carcinoma | 78 | 84.6% | 26.9% | NA | NA | **TRAEs:** skin (43.6%), endocrine (11.5%),  gastrointestinal (14.1%), renal (9.0%),  hepatic (7.0%),  pulmonary (2.6%) | **TRAEs:** skin (3.8%), renal (2.6%), hepatic (2.6%), gastrointestinal (1.3%),  pneumonitis (1.3%) | NA | Sharma et al., 2019 |
|  |  |  | esophagogastric cancer | 59 | 69.0% | 17.0% | NA | NA | **TRAEs:** fatigue (34%),  pruritus (17%), diarrhea (15%), decreased appetite (15%), AST increased (12%), ALT increased (8%) | **TRAEs:** AST increased (5%),  ALT increased (3%),  diarrhea (2%),  fatigue (2%) | NA | Janjigian et al., 2018 |
|  | KEYNOTE028 (NCT02054806) | pembrolizumab 10 mg/kg | small cell lung cancer | 24 | 66.7% | 8.3% | NA | NA | **TRAEs:** arthralgia (16.6%),  asthenia (16.6%),  rash (16.7%),  diarrhea (12.5%),  fatigue (12.5%) | **TRAEs:** grade 3 bilirubin elevation (4.2%),  grade 5 colitis/intestinal ischemia (4.2%) | colitis and intestinal ischemia (4.2%) | Ott et al., 2017b |
|  |  |  | malignant pleural  mesothelioma | 25 | 64.0% | 20.0% | 12.0% | NA | **TRAEs:** infusion-related reaction (4%), hypothyroidism (4%), rhabdomyolysis (4%), erythema (4%), erythema multiforme (4%), iridocyclitis (4%), | **TRAEs:** grade 3 rhabdomyolysis (4%), grade 3 erythema (4%), grade 3 iridocyclitis (4%) | none | Alley et al., 2017 |
|  |  |  | advanced salivary gland carcinoma | 26 | 85.0% | 12.0% | 23.0% | 7.7% | **TRAEs:** hypothyroidism (15%),  hepatitis (3.8%),  interstitial lung disease (3.8%) | **TRAEs:** grade 3 hepatitis (3.8%),  grade 5 interstitial lung disease (3.8%) | none | Cohen et al., 2018 |
|  |  |  | advanced carcinoid or pancreatic neuroendocrine (pNET) | carcinoid n=25, pNET n=16 | **carcinoid** 68% **pNET** 68.8% | **carcinoid** 32% **pNET** 6.3% | **carcinoid** 36% **pNET** 19% | NA | **carcinoid TRAE:** diarrhea (28%),  fatigue (24%),  hypothyroidism (16%), **pNET TRAEs:** fatigue (37.5%), pruritus (18.8%), hypothyroidism (12.5%),  rash (12.5%), myalgia (12.5%) | **carcinoid TRAEs:**  diarrhea (12%),  AST increase (8%), ALT increase (8%), **pNET TRAEs:** fatigue (6.3%) | none | Mehnert et al., 2020 |
|  |  |  | nasopharyngeal carcinoma | 27 | 74.1% | 29.6% | 37.0% | NA | **irAEs:**hepatitis (14.8%),  hypothyroidism (7.4%),  pneumonitis (7.4%). **TRAEs:**  rash (25.9%), pruritus(25.9%), pain (22.2%), hypothyroidism (18.5%), fatigue (18.5%), | **TRAEs:** hepatitis (7.4%), pneumonitis (7.4%),  anemia (3.7%),  sepsis (3.7%), blood creatine  phosphokinase level increased (3.7%),  proteinuria (3.7%) | sepsis (3.7%) | Hsu et al., 2017 |
|  |  |  | recurrent carcinoma of the anal canal | 43 | 64.0% | 16.0% | NR | NA | **TRAEs:** diarrhea (16%),  fatigue (16%),  nausea (12%) | **TRAEs:** colitis (4%),  diarrhea (4%),  general physical health deterioration (1%), increased blood thyroid  stimulating hormone (1%), | none | Ott et al., 2017c |
|  |  |  | cervical cancer | 24 | 75.0% | 21.0% | 25.0% | NA | **IRAEs:** rash (8.3%),  colitis (4.2%),  Guillain-Barré syndrome (4.2%), hyperthyroidism (4.2%), hypothyroidism (4.2%) | NR | none | Jean-Sebastien et al., 2017; |
|  |  |  | advanced colorectal carcinoma | 137 | 35.0% | 4.0% | 4.0% | NA | **TRAEs:**  fatigue (13%),  asthenia (9%), stomatitis (9%) | **TRAEs:** increased blood bilirubin (4%) | none | O'Neil et al., 2017 |
|  |  |  | advanced breast cancer | 25 | 64.0% | 16.0% | 20.0% | 4.0% | **IRAEs:** grade 2 hypothyroidism (1%),  grade 2 hyperthyroidism (1%), grade 2 infusion related reaction (1%), grade 1 pneumonitis (1%) grade 3 autoimmune hepatitis (1%) | **IRAEs:** grade 3 autoimmune hepatitis (1%) | none | Hope et al., 2018 |
|  |  |  | endometrial cancer | 75 | 54.2% | 5.3% | NA | NA | **TRAEs:**  fatigue (20.8%), pruritus (16.7%), pyrexia (12.5%), decreased appetite (12.5%) | **TRAEs:** one diarrhea (1%), one chills and pyrexia (1%), one anemia, hyperglycemia,  and hyponatremia (1%), one asthenia and back pain (1%) | none | Ott et al., 2017a |
|  |  |  | papillary or follicular thyroid cancer | 22 | 82.0% | 4.5% | NA | NA | **TRAEs:** diarrhea (32%), fatigue (18%), pruritus (14%), rash (14%) | **TRAEs:** colitis (4.5%) | none | Mehnert et al., 2019 |
|  |  |  | advanced prostate adenocarcinoma | 23 | 60.9% | 13.0% | NA | NA | **TRAEs:** nausea (13%),  hyperthyroidism (8.7%), fatigue (8.7%), diarrhea (8.7%), pruritus (8.7%) | **TRAEs:** asthenia (4.3%), fatigue (4.3%), increased lipase (4.3%), peripheral neuropathy (4.3%) | none | Hansen et al., 2018 |
|  |  |  | advanced biliary cancer | 24 | 66.7% | 16.7% | 16.7% | 8.3% | **IRAEs:** hypothyroidism (8.3%), severe skin reaction (4.2%), infusion reaction (4.2%) | **IRAEs:** severe skin reaction (4.2%), colitis (4.2%) | none | Piha-Paul et al.,2020 |
|  | KEYNOTE158 (NCT02628067) | pembrolizumab 200mg | small cell lung cancer | 107 | 60.0% | 12.0% | 33.0% | 5.0% | **IRAEs:** hypothyroidism (12%),  hyperthyroidism (7%),  severe skin reactions (3%),  adrenal insufficiency (2%), nephritis (2%),  pneumonitis (2%), pancreatitis (2%) | **IRAEs：** severe skin reactions (1%), adrenal insufficiency (1%), pancreatitis (2%), pneumonitis (1%), colitis (1%） | pneumonitis (0.9%),  encephalopathy (0.9%) | Chung et al., 2020 |
|  |  |  | advanced cervical cancer | 98 | 65.3% | 12.2% | 25.5% | 5.1% | **IRAEs:** hypothyroidism (11.2%), hyperthyroidism (9.2%), infusion-related reaction (3.1%), colitis (2%), hepatitis (2%), severe skin reactions (2%) | **IRAEs:** hepatitis (2%), severe skin reactions (2%), adrenal insufficiency (1%) | none | Chung et al., 2019 |
|  |  |  | advanced neuroendocrine tumors | 107 | 75.7% | 20.6% | 22.4% | 8.4% | **IRAEs:** hypothyroidism (10.3 %), hyperthyroidism (3.7%), pneumonitis (2.8%), hepatitis (2.8%), severe skin reactions (2.8%) | **IRAEs:** hepatitis (2.8%), severe skin reactions (2.8%), colitis (1.9%), adrenal insufficiency (0.9%) | autoimmune hepatitis (0.9%) | Strosberg et al., 2020 |
|  |  |  | advanced biliary cancer | 104 | 54.8% | 13.5% | 18.3% | 5.8% | **IRAEs:** hypothyroidism (7.7%), pneumonitis (5.8%), severe skin reaction (2.9%), hepatitis (2.9%), | **IRAEs:** severe skin reaction (1.9%), colitis (1.0%), pneumonitis (1.0%), hepatitis (1.0%), myositis (1.0%), type 1 diabetes mellitus (1.0%) | renal failure (1.0%) | Piha-Paul et al.,2020 |
| PD-L1 | NCT01375842 | atezolizumab 15 mg/kg or 1200 mg | small cell lung cancer | 17 | 65.0% | 17.6% | NA | NA | **TRAEs:** fatigue (24%) | **TRAEs:** pneumonitis (5.9%), hepatic failure (5.9%) | none | Sequist et al., 2016 |
|  |  |  | metastatic urothelial cancer | 95 | 67.0% | 9.0% | NA | NA | **TRAEs:** fatigue (18%), asthenia (14%), decreased appetite (13%), pruritus (13%), nausea (12%), rash (8%) | **TRAEs:** asthenia (2%), aspartate aminotransferase level  increased (2%) | none | Petrylak et al., 2018 |
|  |  |  | head and neck cancer | 32 | 66.0% | 13.0% | NA | NA | **TRAEs:** fatigue (22%), rash (16%), | **TRAEs:** tumor lysis syndrome (6%), colitis (3%), hyponatremia (3%), pruritus (3%), cardiac tamponade (3%) | none | D et al., 2018 |
|  |  |  | metastatic renal cell carcinoma | 70 | 84.0% | 17.0% | 43.0% | 4.2% | **TRAEs:** fatigue (41%), cough (36%), arthralgia (30%), | **TRAEs:** anemia (7%), dyspnea (7%), | NA | Mcdermott et al., 2016 |
|  |  |  | metastatic triple-negative breast cancer | 116 | 63.0% | 11.0% | NA | NA | **TRAEs:** pyrexia (16%),  fatigue (13%), nausea (11%),  diarrhea (10%),  asthenia (10%),  pruritus (10%) | **TRAEs:** hyperglycemia (0.8%), pneumonitis (0.8%) | NA | Emens et al., 2018 |
|  | NCT03432598 | tislelizumab 200 mg + etoposide-platinum | small cell lung cancer | 17 | 100.0% | 76.5% | 35.3% | none | **IRAEs:** thyroid disorders (29.4%), pneumonitis (5.9%), type 1 diabetes mellitus (5.9%) | none | none | Wang et al., 2020b |
|  |  |  | nonsquamous non-small cell lung cancer | 16 | 100.0% | 68.8% | 12.5% | none | **IRAEs:** thyroid disorders (6.3%), pneumonitis (6.3%) | none | none | Wang et al., 2020b |
|  |  |  | squamous NSCLC | 21 | 100.0% | 71.4% | 52.4% | 14.3% | **IRAEs:** thyroid disorders (14%), pneumonitis (9.5%), hepatitis (19%), myositis (9.5%) | **IRAEs:** hepatitis (9.5%), myositis (4.8%) | none | Wang et al., 2020b |
|  |  |  |  |  |  |  |  |  |  |  |  |  |

Abbreviations: AST: aspartate aminotransferase; ALT: alanine transaminase; NA: not available

**REFERENCES**

Alley, E.W., Lopez, J., Santoro, A., Morosky, A., Saraf, S., Piperdi, B., and Brummelen, E.V. (2017). Clinical safety and activity of pembrolizumab in patients with malignant pleural mesothelioma (KEYNOTE-028): preliminary results from a non-randomised, open-label, phase 1b trial. The Lancet Oncology.

Chung, H., Piha-Paul, S., Lopez-Martin, J., Schellens, J., Kao, S., Miller, W., Delord, J., Gao, B., Planchard, D., Gottfried, M.*, et al.* (2020). Pembrolizumab After Two or More Lines of Previous Therapy in Patients With Recurrent or Metastatic SCLC: Results From the KEYNOTE-028 and KEYNOTE-158 Studies. Journal of thoracic oncology : official publication of the International Association for the Study of Lung Cancer *15*, 618-627.

Chung, H.C., Ros, W., Delord, J.P., Perets, R., Italiano, A., Shapira-Frommer, R., Manzuk, L., Piha-Paul, S.A., Xu, L., and Zeigenfuss, S. (2019). Efficacy and Safety of Pembrolizumab in Previously Treated Advanced Cervical Cancer: Results From the Phase II KEYNOTE-158 Study. Journal of Clinical Oncology.

Cohen, R.B., Delord, J.P., Doi, T., Piha-Paul, S.A., Liu, S.V., Gilbert, J., Algazi, A.P., Damian, S., Hong, R.L., and Le Tourneau, C. (2018). Pembrolizumab for the Treatment of Advanced Salivary Gland Carcinoma: Findings of the Phase 1b KEYNOTE-028 Study. American journal of clinical oncology, 1.

D, A., Colevas, Bahleda, Braiteh, Balmanoukian, Brana, G, N., Chau, Sarkar, and Molinero (2018). Safety and Clinical Activity of Atezolizumab in Head and Neck Cancer: Results From a Phase I Trial. Annals of oncology : official journal of the European Society for Medical Oncology.

Emens, L.A., Cruz, C., Eder, J.P., Braiteh, F., and Schmid, P. (2018). Long-term Clinical Outcomes and Biomarker Analyses of Atezolizumab Therapy for Patients With Metastatic Triple-Negative Breast Cancer: A Phase 1 Study. JAMA oncology 5.

Hansen, A.R., Massard, C., Ott, P.A., Haas, N.B., Lopez, J.S., Ejadi, S., Wallmark, J.M., Keam, B., J-P, D., and Aggarwal, R. (2018). Pembrolizumab for advanced prostate adenocarcinoma: findings of the KEYNOTE-028 study. Annals of Oncology *29*, 1807-1813.

Hope, Rugo, Jean-Pierre, Delord, Seock-Ah, Im, Patrick, and Ott (2018). Safety and Antitumor Activity of Pembrolizumab in Patients with Estrogen Receptor?Positive/Human Epidermal Growth Factor Receptor 2?Negative Advanced Breast Cancer. Clinical Cancer Research An Official Journal of the American Association for Cancer Research.

Hsu, C., Lee, S.H., Ejadi, S., Even, C., Cohen, R.B., Le Tourneau, C., Mehnert, J.M., Algazi, A., Van Brummelen, E.M.J., and Saraf, S. (2017). Safety and Antitumor Activity of Pembrolizumab in Patients With Programmed Death-Ligand 1–Positive Nasopharyngeal Carcinoma: Results of the KEYNOTE-028 Study. Journal of Clinical Oncology *35*, 4050-4056.

Janjigian, Y., Bendell, J., Calvo, E., Kim, J., Ascierto, P., Sharma, P., Ott, P., Peltola, K., Jaeger, D., Evans, J.*, et al.* (2018). CheckMate-032 Study: Efficacy and Safety of Nivolumab and Nivolumab Plus Ipilimumab in Patients With Metastatic Esophagogastric Cancer. Journal of clinical oncology : official journal of the American Society of Clinical Oncology *36*, 2836-2844.

Jean-Sebastien, Frenel, Christophe, Le, Tourneau, Bert, O’Neil, Patrick, A., and Ott (2017). Safety and Efficacy of Pembrolizumab in Advanced, Programmed Death Ligand 1–Positive Cervical Cancer: Results From the Phase Ib KEYNOTE-028 Trial. Journal of Clinical Oncology.

Mcdermott, D.F., Sosman, J.A., Sznol, M., Massard, C., Gordon, M.S., Hamid, O., Powderly, J.D., Infante, J.R., Fassò, M., and Wang, Y.V. (2016). Atezolizumab, an Anti–Programmed Death-Ligand 1 Antibody, in Metastatic Renal Cell Carcinoma: Long-Term Safety, Clinical Activity, and Immune Correlates From a Phase Ia Study. Journal of Clinical Oncology.

Mehnert, J., Bergsland, E., O'Neil, B., Santoro, A., Schellens, J., Cohen, R., Doi, T., Ott, P., Pishvaian, M., Puzanov, I.*, et al.* (2020). Pembrolizumab for the treatment of programmed death-ligand 1-positive advanced carcinoid or pancreatic neuroendocrine tumors: Results from the KEYNOTE-028 study. Cancer *126*, 3021-3030.

Mehnert, J.M., Varga, A., Brose, M.S., Aggarwal, R.R., Lin, C.C., Prawira, A., De Braud, F., Tamura, K., Doi, T., and Piha-Paul, S.A. (2019). Safety and antitumor activity of the anti–PD-1 antibody pembrolizumab in patients with advanced, PD-L1–positive papillary or follicular thyroid cancer. BMC Cancer *19*.

O'Neil, B.H., Wallmark, J.M., Lorente, D., Elez, E., and Han, S.W. (2017). Safety and antitumor activity of the anti–PD-1 antibody pembrolizumab in patients with advanced colorectal carcinoma. PloS one *12*, e0189848.

Ott, P., Bang, Y., Berton-Rigaud, D., Elez, E., Pishvaian, M., Rugo, H., Puzanov, I., Mehnert, J., Aung, K., Lopez, J.*, et al.* (2017a). Safety and Antitumor Activity of Pembrolizumab in Advanced Programmed Death Ligand 1-Positive Endometrial Cancer: Results From the KEYNOTE-028 Study. Journal of clinical oncology : official journal of the American Society of Clinical Oncology *35*, 2535-2541.

Ott, P.A., Elez, E., Hiret, S., Kim, D.W., Morosky, A., Saraf, S., Piperdi, B., and Mehnert, J.M. (2017b). Pembrolizumab in Patients With Extensive-Stage Small-Cell Lung Cancer: Results From the Phase Ib KEYNOTE-028 Study. Journal of Clinical Oncology Official Journal of the American Society of Clinical Oncology *35*, JCO2017725069.

Ott, P.A., Piha-Paul, S.A., Munster, P., Pishvaian, M.J., Van Brummelen, E.M.J., Cohen, R.B., Gomez-Roca, C., Ejadi, S., Stein, M., and Chan, E. (2017c). Safety and antitumor activity of the anti-PD-1 antibody pembrolizumab in patients with recurrent carcinoma of the anal canal. Annals of Oncology *28*, 1036-1041.

Piha-Paul, S., Oh, D., Ueno, M., Malka, D., Chung, H., Nagrial, A., Kelley, R., Ros, W., Italiano, A., Nakagawa, K.*, et al.* (2020). Efficacy and safety of pembrolizumab for the treatment of advanced biliary cancer: Results from the KEYNOTE-158 and KEYNOTE-028 studies. International journal of cancer *147*, 2190-2198.

Piha-Paul, S., Oh, D., Ueno, M., Malka, D., Chung, H., Nagrial, A., Kelley, R., Ros, W., Italiano, A., Nakagawa, K., et al. (2020). Efficacy and safety of pembrolizumab for the treatment of advanced biliary cancer: Results from the KEYNOTE-158 and KEYNOTE-028 studies. International journal of cancer 147, 2190-2198.

Ready, N., Farago, A., de Braud, F., Atmaca, A., Hellmann, M., Schneider, J., Spigel, D., Moreno, V., Chau, I., Hann, C.*, et al.* (2019). Third-Line Nivolumab Monotherapy in Recurrent SCLC: CheckMate 032. Journal of thoracic oncology : official publication of the International Association for the Study of Lung Cancer *14*, 237-244.

Sequist, L.V., Chiang, A., Gilbert, J., Gordon, M., and Gettinger, S. (2016). Clinical activity, safety and predictive biomarkers results from a phase Ia atezolizumab (atezo) trial in extensive-stage small cell lung cancer (ES-SCLC). Annals of Oncology 27.

Sharma, P., Siefker-Radtke, A., de Braud, F., Basso, U., Calvo, E., Bono, P., Morse, M., Ascierto, P., Lopez-Martin, J., Brossart, P.*, et al.* (2019). Nivolumab Alone and With Ipilimumab in Previously Treated Metastatic Urothelial Carcinoma: CheckMate 032 Nivolumab 1 mg/kg Plus Ipilimumab 3 mg/kg Expansion Cohort Results. Journal of clinical oncology : official journal of the American Society of Clinical Oncology *37*, 1608-1616.

Strosberg, J.R., Mizuno, N., Doi, T., Grande, E., and Hadoux, J. (2020). Efficacy and Safety of Pembrolizumab in Previously Treated Advanced Neuroendocrine Tumors: Results From the Phase 2 KEYNOTE-158 Study. Clinical Cancer Research, clincanres.3014.2019.

Wang, Z., Zhao, J., Ma, Z., Cui, J., Shu, Y., Liu, Z., Cheng, Y., Leaw, S., Wu, Y., Ma, Y.*, et al.* (2020b). A Phase 2 Study of Tislelizumab in Combination With Platinum-Based Chemotherapy as First-line Treatment for Advanced Lung Cancer in Chinese Patients. Lung cancer (Amsterdam, Netherlands) *147*, 259-268.
